# Development of Halogenated Pyrazolines as Selective Monoamine Oxidase-B Inhibitors: Deciphering via Molecular Dynamics Approach

**DOI:** 10.3390/molecules26113264

**Published:** 2021-05-28

**Authors:** Aathira Sujathan Nair, Jong-Min Oh, Vishal Payyalot Koyiparambath, Sunil Kumar, Sachithra Thazhathuveedu Sudevan, Opeyemi Soremekun, Mahmoud E. Soliman, Ahmed Khames, Mohamed A. Abdelgawad, Leena K. Pappachen, Bijo Mathew, Hoon Kim

**Affiliations:** 1 Department of Pharmaceutical Chemistry, Amrita School of Pharmacy, AIMS Health Sciences Campus, Amrita Vishwa Vidyapeetham, Kochi 682 041, India; sujuaathira@gmail.com (A.S.N.); vishalpk11@gmail.com (V.P.K.); solankimedchem@gmail.com (S.K.); sachithrasudevan22@gmail.com (S.T.S.); 2 Department of Pharmacy, Research Institute of Life Pharmaceutical Sciences, Sunchon National University, Suncheon 57922, Korea; ddazzo005@naver.com; 3 Molecular Bio-computation and Drug Design Laboratory, School of Health Sciences, Westville Campus, University of KwaZulu-Natal, Durban 4001, South Africa; opeyemisoremekun@gmail.com (O.S.); soliman@ukzn.ac.za (M.E.S.); 4 Department of Pharmaceutics and Industrial Pharmacy, College of Pharmacy, Taif University, P.O. Box-11099, Taif 21944, Saudi Arabia; a.khamies@tu.edu.sa; 5 Department of Pharmaceutical Chemistry, College of Pharmacy, Jouf University, Sakaka 72341, Saudi Arabia; hmdgwd@ju.edu.sa; 6 Department of Pharmaceutical Organic Chemistry, Faculty of Pharmacy, Beni-Suef University, Beni Suef 62514, Egypt

**Keywords:** halogenated pyrazolines, monoamine oxidase inhibitors, kinetics, reversibility, molecular dynamics

## Abstract

Halogens have been reported to play a major role in the inhibition of monoamine oxidase (MAO), relating to diverse cognitive functions of the central nervous system. Pyrazoline/halogenated pyrazolines were investigated for their inhibitory activities against human monoamine oxidase-A and -B. Halogen substitutions on the phenyl ring located at the fifth position of pyrazoline showed potent MAO-B inhibition. Compound 3-(4-ethoxyphenyl)-5-(4-fluorophenyl)-4,5-dihydro-1*H*-pyrazole (**EH7**) showed the highest potency against MAO-B with an IC_50_ value of 0.063 µM. The potencies against MAO-B were increased in the order of –F (in **EH7**) > –Cl (**EH6**) > –Br (**EH8**) > –H (**EH1**). The residual activities of most compounds for MAO-A were > 50% at 10 µM, except for **EH7** and **EH8** (IC_50_ = 8.38 and 4.31 µM, respectively). **EH7** showed the highest selectivity index (SI) value of 133.0 for MAO-B, followed by **EH6** at > 55.8. **EH7** was a reversible and competitive inhibitor of MAO-B in kinetic and reversibility experiments with a Ki value of 0.034 ± 0.0067 µM. The molecular dynamics study documented that **EH7** had a good binding affinity and motional movement within the active site with high stability. It was observed by MM-PBSA that the chirality had little effect on the overall binding of **EH7** to MAO-B. Thus, **EH7** can be employed for the development of lead molecules for the treatment of various neurodegenerative disorders.

## 1. Introduction

Monoamine oxidases (MAOs) are the principal metabolizing enzymes responsible for the oxidative degradation of various biogenic amines that are related to diverse cognitive functions of the central nervous system (CNS) [1]. Considering the difference in the structure, substrate specificity, biological functions, and catalytic mechanism of MAOs, two types of isoforms are available, namely, MAO-A and MAO-B [2]. Ammonia, aldehyde, and hydrogen peroxide are the major intermediate products formed during MAO-based catalyzed oxidative deamination [3]. These can produce substantial issues, such as astrocyte swelling, unbalancing of the signaling process in the neurotransmission, neuronal loss, and mitochondrial dysfunctions [4]. Eventually, these toxic by-products can lead to numerous neurodegenerative disorders, such as Alzheimer’s disease (AD) and Parkinson’s disease (PD) [5]. Selegiline is a selective/irreversible MAO-B inhibitor used as an adjuvant therapy with _L_-DOPA, which is considered a safe therapy for PD [6,7]. The latest research has also documented that potent, reversible dual targeted MAO-B/AChE inhibitors can decrease the production of β-amyloid peptide levels in AD-related neurons [8]. In 2017, safinamide, as the first selective and reversible MAO-B inhibitor approved by the USFDA for PD treatment, along with levodopa, has promising neuroprotective effects on MPTP-treated mice [9,10].

**Figure 1:**
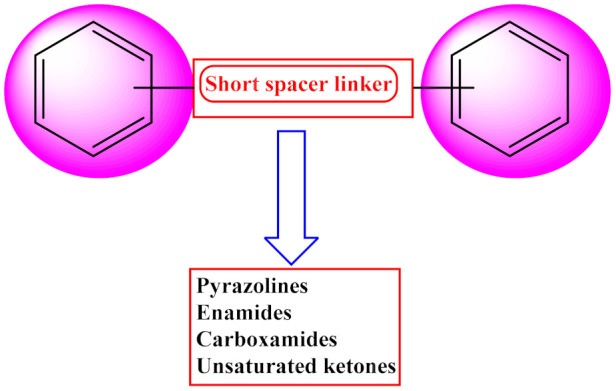
General blueprint of the design of MAO-B inhibitors.

In the exploration of novel, selective, and reversible MAO-B inhibitors, a general blueprint of drug design has been widely accepted recently, and it consists of a molecular framework of two hydrophobic rings of phenyl/heteroaryl, which are separated by an electron-rich and flexible short spacer unit (Figure 1) [11]. Many of the molecules from this diverse class, such as pyrazolines, enamides, carboxamides, and α, β-unsaturated ketones, were identified as potent MAO-B inhibitors [12,13,14,15,16,17,18,19,20,21]. The presence of a rotatable bond and electron-rich Michael acceptor unit in the spacer can efficiently make appropriate orientations and binding affinities in the entrance and substrate cavity of MAO-B. The flexibility of the molecules provides good recognition of aromatic systems of the ligand to the hydrophobic cavity of the inhibitor binding cavity of MAO-B by Pi–Pi stacking or Pi–cation interactions. The binding requirements of the selective MAO-B inhibitors include one or more hydrophobic ring, preferably an aromatic or heteroaromatic nucleus with small lipophilic substituents, such as dimethylamino, ethoxy, methoxy, and halogens. The presence of a hydrogen bond acceptor (HBA) and hydrogen bond donor (HBD) linker with optimal rotatable bonds between these hydrophobic units had a pivotal role in the design of potent MAO-B inhibitors [22,23,24,25,26].

Pyrazoline, or dihydropyrazole, is the partially reduced form of pyrazole, and 2-pyrazoline is the most stable tautomeric from in this class [27]. The presence of an asymmetric carbon atom at the C5 position of pyrazoline was revealed to be due to two epimers of the *R* and *S* forms [28]. The ring closure reaction of α, β-unsaturated ketones with hydrazine derivatives in the presence of basic medium is the commonly used synthetic methodology for 2-pyrazolines [29]. The separation of the *R* and *S* forms from the racemic mixture of 2-pyrazoline is a considerable task in the identification of biologically more active isomers [30]. Many studies of the literature have documented that the substitution of the 3rd and 5th position of pyrazolines with aryl/heteroaryl units can favor MAO-B inhibition [31]. At the same time, the length and bulkiness of the groups anchoring from the N1 position of pyrazolines can shift the selectivity from MAO-B to MAO-A. The presence of lipophilic groups, such as halogens, hydroxyl, and methoxy groups, on the phenyl ring of the third and fifth positions of the pyrazoline nucleus, can cause significant MAO-B inhibition [32,33,34,35].

Regarding the role of halogens in MAO inhibition, it is generally known that FDA-approved MAO inhibitors like clorgyline, moclobemide, safinamide, and lazabemide are chlorine-containing drugs [36]. The evidence shows that more than 40% of drugs in FDA-approved or clinical trials are in the halogenated form [37]. Recently, our group emphasized the presence and orientation of various halogens on chemical scaffolds, such as chalcones, chromones, coumarins, hydrazothiazoles, and pyrazolines, thereby providing in-depth knowledge on MAO inhibition and B isoform selectivity. It was noted that this can have a new type of impact on the drug–target interaction of these MAO inhibitors in order to fill the small lipophilic pockets in the inhibitor binding cavity (IBC) of MAOs [38].

The objective of the present study was focused on the in vitro evaluation of MAO-A and MAO-B inhibitions of pyrazoline/halogenated pyrazoline derivatives. The lead molecules were further studied for kinetics, reversibility, and blood–brain barrier (BBB) permeation. Detailed molecular dynamics established precisely the protein–ligand binding interactions of the halogenated molecules in their respective active sites of enzyme targets and the role of chirality towards MAO-B inhibition.

## 2. Results and Discussion

### 2.1. Inhibitory Activities against MAO Enzymes

At 10 µM, all derivatives in the **EH** sequence had potent inhibitory action against MAO-B, with residual activities of about 50% (Table 1). Fluorine-containing compound **EH7** showed the greatest inhibitory activities against MAO-B with an IC_50_ value of 0.063 µM, followed by chlorine-containing **EH6** (IC_50_ = 0.40 µM), and bromine-containing **EH8** (IC_50_ = 0.69 µM). Comparing **EH1** (IC_50_ = 5.38 µM) with **EH7**, the potencies for MAO-B greatly increased, i.e., by 85.4 times, based on their IC_50_ values, with the presence of –F instead of –H. Most of the compounds weakly inhibited MAO-A at 10 µM, with residual activities of >50%, except **EH7** and **EH8**. Among them, **EH7** showed the highest selectivity index (SI) value for MAO-B with 133.0, followed by **EH6** with 55.8.

The enzyme inhibition results obtained in the current study revealed that all of the halogenated pyrazoline derivatives showed good selective MAO-B inhibitory activity. The study mainly confirmed the effect of halogens on the *para*-position of the **B** ring of ethoxylated pyrazolines. Among the halogens, fluorine had the greatest impact on MAO-B inhibition with a sub-micromolar level range. It is worth noting that a highly electronegative fluorine atom-containing compound is two times more potent than marketed drugs for the inhibition of MAO-B, such as reversible-type lazabemide (IC_50_ = 0.11 µM) and irreversible pargyline (IC_50_ = 0.14 µM). Recent reports state that the anisotropy of charge circulation around the fluorine atom, which is the reason for the directional and stabilizing property of the active site, proves that halogen enhances the analogy of drugs of new molecules [38].

Recently, we evaluated MAO-B inhibitory activities for ethoxylated head of chalcone derivatives and found that chlorine-, fluorine-, or bromine-containing chalcones had good IC_50_ values of 0.57, 0.053, and 2.01 µM, respectively [39]. Cyclization with hydrazine hydrate of the same halogenated molecules retained higher MAO-B inhibitory activities than the unsubstituted one. This finding clearly shows the importance of halogen incorporation both in ethoxylated chalcones and their pyrazolines moieties for MAO-B inhibition. Interestingly, in both series, fluorine-containing compounds showed dominant MAO-B inhibition profiles. For the purpose of the development of compounds with good MAO-B inhibitory activity, the bonding of fluorine to the molecule was an auspicious lead.

### 2.2. Kinetics of MAO Inhibition

**Figure 2:**
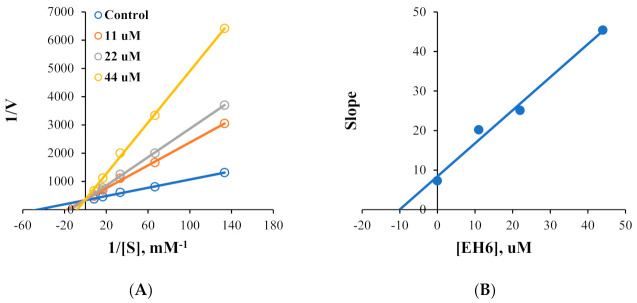

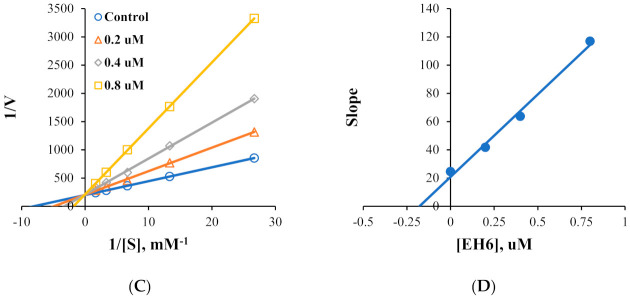
Lineweaver–Burk plots for MAO-A and MAO-B inhibitions by **EH6** (**A**,**C**) and their respective secondary plots (**B**,**D**) of slopes vs. inhibitor concentrations.

**Figure 3:**
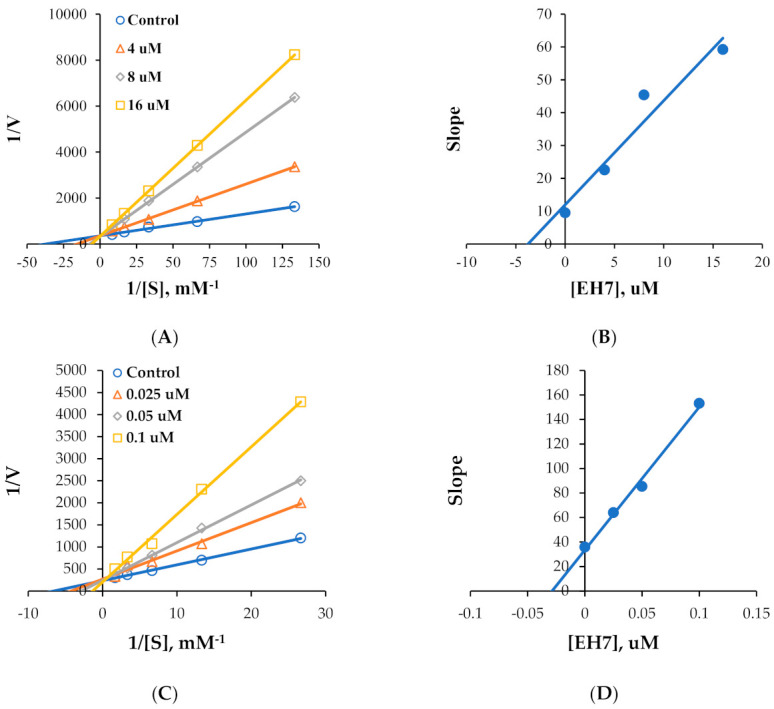
Lineweaver–Burk plots for MAO-A and MAO-B inhibitions by **EH7** (**A**,**C**) and their respective secondary plots (**B**,**D**) of slopes vs. inhibitor concentrations.

Kinetic studies on MAO-A and MAO-B inhibitions by **EH6** and **EH7** were performed. Lineweaver–Burk plots and secondary plots showed that **EH6** was a competitive inhibitor of MAO-A and MAO-B (Figure 2A,C), with K_i_ values of 10.8 ± 1.03 and 0.19 ± 0.016 µM, respectively (Figure 2B,D). Lineweaver–Burk plots and secondary plots exhibited that **EH7** was a competitive inhibitor of MAO-A and MAO-B (Figure 3A,C), with K_i_ values of 3.64 ± 0.18 and 0.034 ± 0.0067 µM, respectively (Figure 3B,D). These results suggest that **EH6** and **EH7** are selective and competitive inhibitors of MAO-B.

### 2.3. Reversibility Study

**Figure 4:**
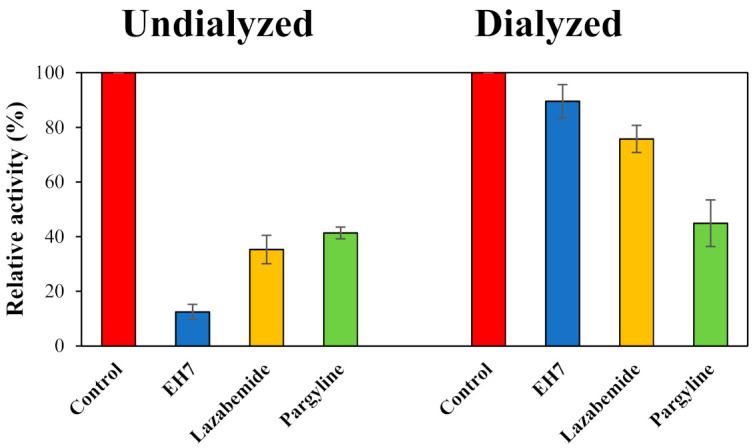
Recovery of MAO-B inhibition by **EH7**, using dialysis experiments. The concentrations used were **EH7** at 0.15 µM, lazabemide (a reversible reference inhibitor) at 0.20 µM, and pargyline (an irreversible reference inhibitor) at 0.30 µM.

Reversibility studies on MAO-B inhibition were carried out on **EH7**. In these experiments, inhibition of MAO-B by **EH7** was recovered from 12.5% (value of A_U_) to 89.5% (value of A_D_). The recovery value was similar to that of the reversible reference lazabemide (from 35.3% to 75.7%); however, it was different from that of the irreversible inhibitor pargyline (from 41.3% to 44.9%), which was not recovered (Figure 4). Similar to the reversible reference level, inhibition of MAO-B by **EH7** was reversed in these experiments, indicating that **EH7** is a reversible inhibitor of MAO-B.

### 2.4. Blood–Brain Barrier (BBB) Permeation Studies

The CNS bioavailability of the molecules was confirmed by a parallel artificial membrane permeability assay (PAMPA) [40]. From the assay, highly effective permeabilities and high CNS bioavailabilities were observed for the halogenated pyrazolines with Pe ranges of 14.13 ~ 14.56 × 10^−6^ cm/s (Table 2). All halogenated substituted derivatives showed higher CNS permeabilities than unsubstituted ones.

### 2.5. Molecular Dynamics Studies

#### 2.5.1. **EH1**, **EH6**, **EH7**, and **EH8** Exert Differential Impacts on the Stability of MAO-A and MAO-B

**Figure 5:**
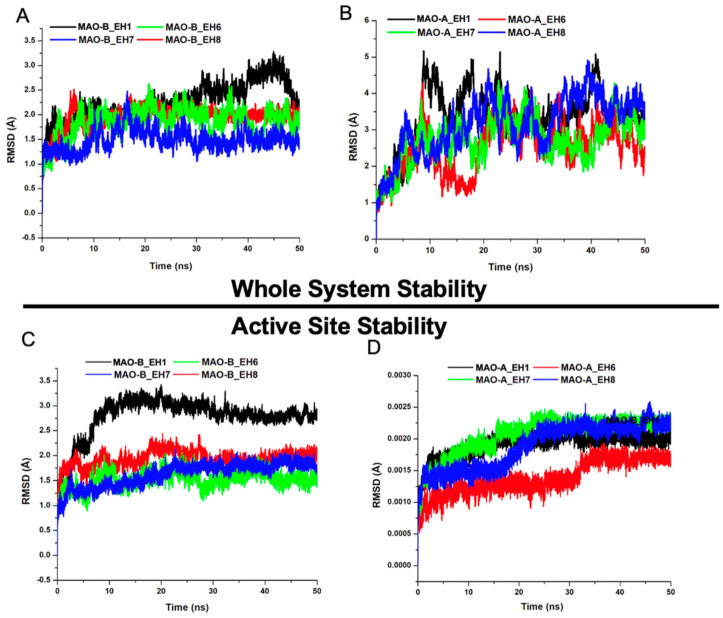
Comparative root mean square deviations (RMSDs) of the Cα carbon atoms of the compounds in whole systems for MAO-B (**A**) and MAO-A (**B**) and in active sites for MAO-B (**C**) and for MAO-A (**D**). (**A**,**C**) **EH1**, black; **EH6**, green; **EH7**, blue; and **EH8** red. (**B**,**D**) **EH1**, black; **EH6**, red; **EH7**, green; and **EH8**, blue.

The root mean square deviation (RMSD) can be used to estimate the conformational stability of a system during a production run [41]. The differential structural perturbatory effect of the ligands on the structure of MAO-B relative to MAO-A was first explored. MAO-B_**EH6**, MAO-B_**EH7**, and MAO-B_**EH8** attained equilibration at around 20 ns (Figure 5). However, MAO-B_**EH1** achieved convergence earlier in the simulation run but eventually elicited a somewhat high structural movement at around 45 ns. In comparison, **EH1**, **EH6**, **EH7**, and **EH8** did not elicit similar stabilizing effects on the MAO-A protein; this could be due to the IC_50_ values experimentally reported, suggesting their low inhibitory potential towards MAO-A. The superior stabilizing effect elicited by the ligands against MAO-B when compared to MAO-A binding points to the high selective targeting of the ligands for MAO-B. As indicated by the IC_50_, **EH7** had the highest stabilizing impact on MAO-B with an average RMSD value of 1.49 Å, while **EH1**, **EH6**, and **EH8** had 2.29, 1.61, and 1.79 Å, respectively (Table 3).

Zeroing on structural influence of the ligands on the mechanistic behavior of the active site could provide more clues regarding their activity. The RMSD of the MAO-A and MAO-B active sites upon **EH1**, **EH6**, **EH7**, and **EH8** binding revealed that **EH6** and **EH7** had the highest active site-stabilizing effect on MAO-B, with overall average RMSD_AS values of 1.61 and 1.49 Å, respectively.

**Figure 6:**
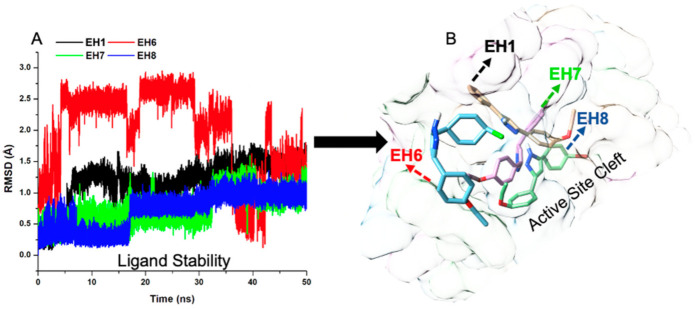
Stability plot of compounds **EH1**, **EH6**, **EH7**, and **EH8**.

The orientation and position of a ligand are considerably indicative of how reactive the ligands can be within the active site. The characteristics of the ligands were therefore investigated. Interestingly, while **EH7** elicited some motional movement within the active site, which made it flexible enough to interact with crucial amino acid residues, it also exhibited good stability—just enough to be adequately anchored for overall binding (Figure 6).

#### 2.5.2. **EH7** Exhibited Low Residual Motion and Trajectory Movement upon Binding to MAO-A and MAO-B

**Figure 7:**
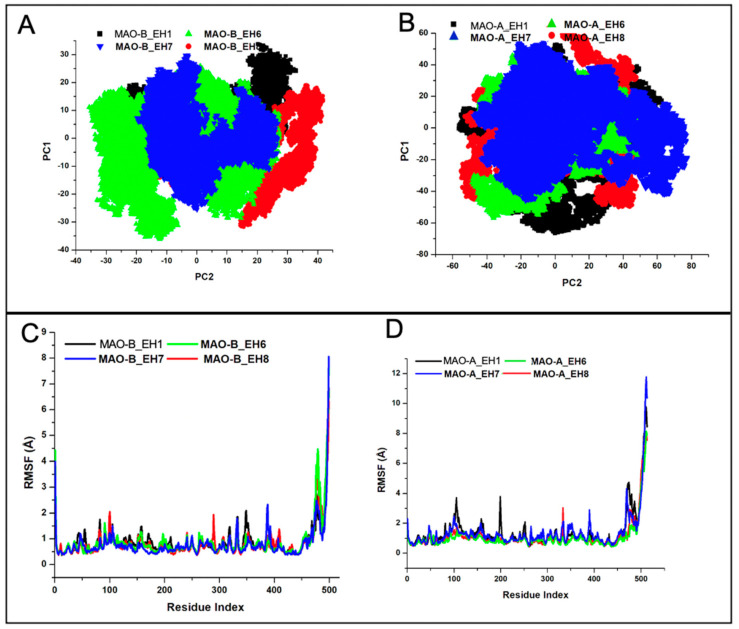
Principal component analysis of the compounds bound to MAO-B (**A**) and to MAO-A (**B**), and root mean square fluctuation (RMSF) of the compounds bound to MAO-B (**C**) and to MAO-A (**D**). (**A**,**B**) **EH1**, black; **EH6**, green; **EH7**, blue; and **EH8**, red. (**C**,**D**) **EH1**, black; **EH6**, green; **EH7**, blue; and **EH8**, red.

Principal component analysis (PCA) is a common molecular dynamic simulation analysis used in the estimation of the essential production run of a system when computed on a low-dimensional free energy space [42]. This estimation is associated with correlated vibrational modes; the translation of the MD trajectory towards the geometric center abolishes the initial rotational and translational movements of the atoms. The decomposition of the trajectory along PC1 and PC2 reveals that upon **EH7** binding to MAO-B, the system exhibits very low diagonalization when compared with other systems (Figure 7A). This further corroborates the results discussed above in the stability section, which attest to the superior activity of **EH7** over other ligands in the series. In contrast, due to the low specificity of **EH1**, **EH6**, **EH7**, and **EH8** towards MAO-A, less high trajectory motion was observed in the ligand-bound MAO-A (Figure 7B).

The root mean square fluctuation (RMSF) computes the residual deviation of a protein over a period of time using a reference position, such as the averaged residual position. RMSF estimates the level of fluctuation of residue from its original position [43]. As depicted in the RMSF plot, the average RMSF values of MAO-B_**EH1**, MAO-B_**EH6**, MAO-B_**EH7**, and MAO-B_**EH8** were 0.88, 0.83, 0.68, and 0.75 Å, respectively (Figure 7C), while those of MAO-A_**EH1**, MAO-A_**EH6**, MAO-A_**EH7**, and MAO-A_**EH8** were 1.47, 1.15, 1.07, and 1.40 Å, respectively (Figure 7D).

#### 2.5.3. Estimation of Free Binding Energies of **EH1**, **EH6**, **EH7**, and **EH8** on MAO-A and MAO-B

The estimation of free binding energy using an MM/PBSA protocol can be employed to gain insight into the models and further obtain the semiquantitative values of their stability. We therefore used a representative snapshot and active site residue decomposition to further explore the time-wise bond interaction occurring between major residues in the active sites of MAO-A/MAO-B and the ligands. MM/PBSA has found useful application in the drug design space used in the estimation of binding affinity between ligands and biomolecules [44].

As indicated in Table 4, **EH7** had higher binding affinity (−36.29 kcal/mol) towards MAO-B than the other ligands, while the binding of **EH1**, **EH6**, and **EH8** was recorded to be −4.11, −11.5, and −5.48 kcal/mol, respectively. Of note is the surprising high binding affinity (−32.55 kcal/mol) of **EH7** for MAO-A. This could be due to the high electronegativity potential of fluorine, which is characteristic of compound **EH7**. In agreement with the IC_50_, the binding affinity of the compounds also followed the same trend.

**Figure 8:**
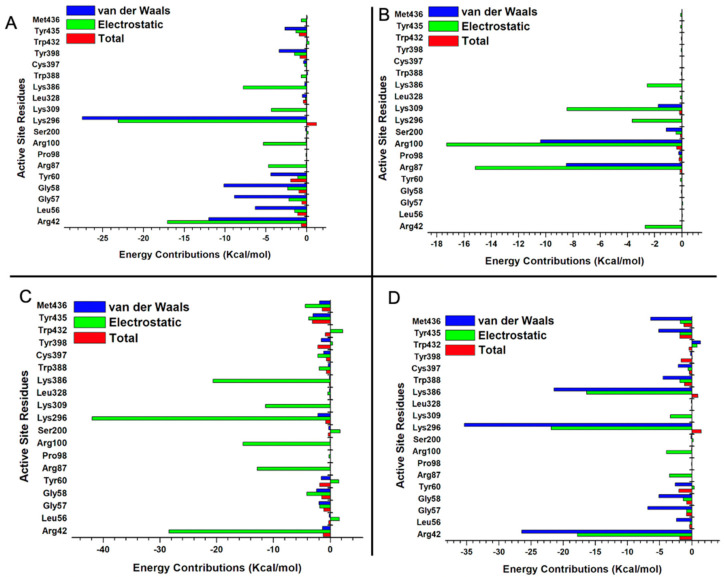
Per residue decomposition of **EH1** bound to MAO-B (**A**), **EH6** bound to MAO-B (**B**), **EH7** bound to MAO-B (**C**), and **EH8** bound to MAO-B (**D**) systems.

**Figure 9:**
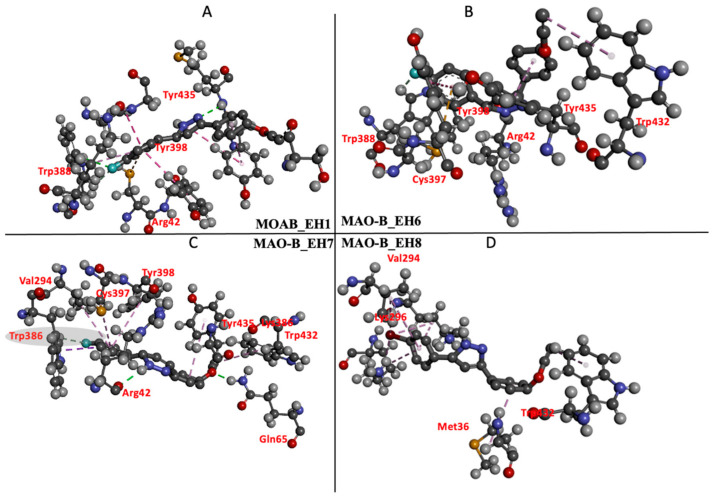
Ligand interaction profile of the interaction of **EH1** (**A**), **EH6** (**B**), **EH7** (**C**), and **EH8** (**D**) with MAO-B.

The individual energy contributions of the active sites were determined using the MM/PBSA per residue energy decomposition protocol [45]. The high binding energy exhibited by **EH7** was contributed by electrostatic interactions of Arg42, Arg87, Arg98, Lys296, and Lys386, with energy values of −28.4, −12.8, −15.3, −41.9, and −20.6 kcal/mol, respectively (Figure 8C). In the interaction between compound **EH6** and MAO-B, residues Arg42, Gly58, Tyr60, Arg87, Pro98, and Arg100 contributed an electrostatic energy of −2.68, −0.04, −0.12, −15.17, −0.14, and −17.27 kcal/mol, respectively (Figure 8B). Despite the electrostatic interactions and other favorable bonds elicited by **EH1**, **EH6**, and **EH8**, compound **EH7** still had the best binding energy and highest contribution of electrostatic interactions. This behavior may not be unrelated to the impact of fluorine found on compound **EH7**. Furthermore, the energetically favored disposition assumed by **EH7** could be attributed to the hydrogen bond formed between the fluorine of **EH7** and hydrogen of Trp386 (Figure 9C). The ligand interaction profile of the interaction of **EH1** (A), **EH6** (B), **EH7** (C), and **EH8** (D) with MAO-B is shown in Figure 9.

#### 2.5.4. Estimation of Free Binding Energies of **EH1**, **EH6**, **EH7**, and **EH8** on MAO-A and MAO-B

**Figure 10:**
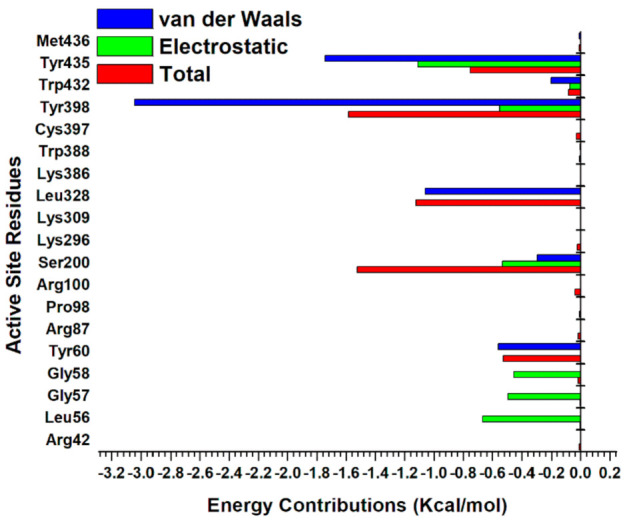
Per residue decomposition of MAO-B_**EH7**S enantiomer.

Since our compounds contain a pyrazoline moiety, it is assumed that they possess an asymmetric carbon atom and, hence, exist as enantiomers. We therefore generated the R and S enantiomers of **EH7** as a representative structure of the compounds, taking into consideration the fact that it has the best IC_50_ and total free energy values. We explored the differential binding and estimated the total free binding energy of the R isomeric form of **EH7** (MAO-B_**EH7**R) and its S counterpart (MAO-B_**EH7**S) using MM/PBSA calculations. Our calculations revealed that MAO-B_**EH7**R and MAO-B_**EH7**S possess binding energies of −36.29 and −36.17 kcal/mol (Table 5). This suggests that the chirality does not change considerably in the overall binding of **EH7** to MAO-B. However, MAO-B_**EH7**S has more van der Waals interactions than MAO-B_**EH7**R. Despite having total free binding energy values that are close, dissimilar residues present in the active sites are responsible for the binding interactions. Chiefly among the residues are Tyr435, Tyr398, and Leu328 (jointly shared by MAO-B_**EH7**R), which contribute van der Waals binding energies of −2.93, −1.58, and −1.00 kcal/mol, respectively (Figure 10).

### 2.6. Target Predicition and ADME Analysis

**Figure 11:**
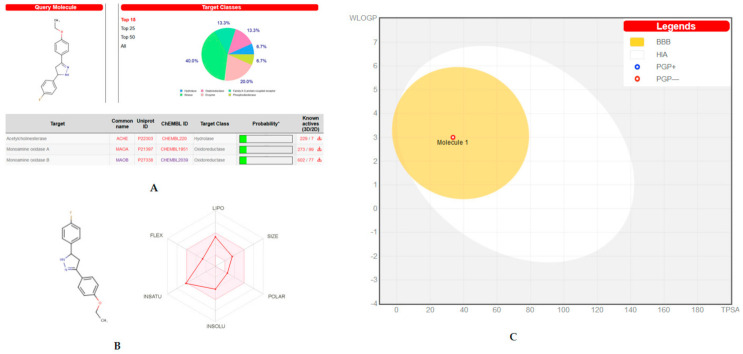
Swiss target/ADME prediction: (**A**) Biological target prediction of lead molecule **EH7**; (**B**) bioavailability radar of **EH7**; (**C**) the BOILED-Egg construction of **EH7**.

The biological target and ADME predictions of the lead molecule of **EH7** were assessed by the online web-based program SwissTarget. The lead molecule showed good prediction results on biological targets of MAO and AChE (Figure 11A). The bioavailability radar gave saturation, size, flexibility, insolubility, and polarity of the molecule, which provided a quick judgement of the drug-likeness of the molecule. The lead molecule was recognized to be within the pink region (optimal ranges) and could be considered a compound endowed with drug-like characteristics (Figure 11B). The BOILED-Egg model also predicted the passive human BBB permeation and gastrointestinal absorption (HIA) for the compound (Figure 11C). The results revealed that **EH7** had comparatively higher probability of BBB and HIA.

## 3. Materials and Methods

### 3.1. Synthesis

**Scheme 1:**
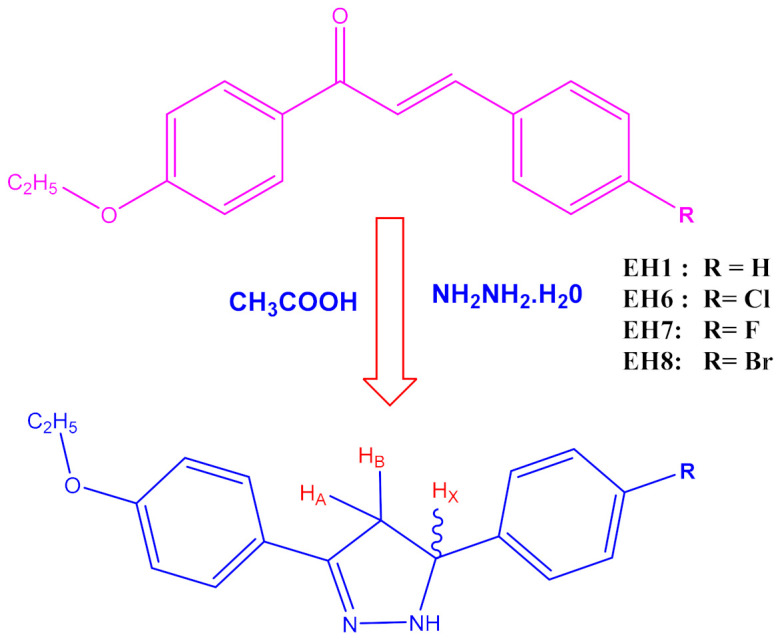
Synthetic route of the compounds under the present study.

A series of ethoxylated chalcone (0.0078 M) was refluxed with (0.0629 M) hydrazine hydrate under ethanol medium. After 14–16 h of reflux, the product was cooled, acidified with dil. HCl, washed thoroughly with water, filtered, dried, and recrystallized from ethanol. The progress of the formation of pyrazoline product was monitored by thin-layer chromatography (TLC; ethylacetate–hexane = 1:9). The synthetic route is depicted in Scheme 1.

*3-(4-ethoxyphenyl)-5-phenyl-4,5-dihydro-1H-pyrazole (**EH1**)*: ^1^H NMR (400 MHz, CDCl_3_) δ: 1.47–1.45 (t, 3H, J = 8.0 Hz, CH_3_), 4.15–4.13 (d, 2H, J = 8.0 Hz, CH_2_), 3.76 (d, 1H, HA), 4.02 (d, 1H, HB), 5.51 (d, 1H, HX), 7.89–7.10 (d, 9H, ArH), 9.21(S, NH). MS (ESI) m/z calcd. for C_17_H_18_N_2_O: 266.3376, found 266.3345.
*5-(4-chlorophenyl)-3-(4-ethoxyphenyl)-4,5-dihydro-1H-pyrazole (**EH6**)*: ^1^H NMR (400 MHz, CDCl_3_) δ: 1.47–1.45 (t, 3H, *J* = 8.0 Hz, CH_3_), 4.14–4.12 (D, 2H, *J* = 8.0 Hz, CH_2_), 3.75 (d, 1H, HA), 4.01 (d, 1H, HB), 5.51 (d, 1H, HX), 7.99–7.08 (d, 8H, ArH) 9.24 (S, NH). MS (ESI) m/z calcd. for C_17_H_17_N_2_OCl: 284.3280, found 284.3276.
*3-(4-ethoxyphenyl)-5-(4-fluorophenyl)-4,5-dihydro-1H-pyrazole (**EH7**):* ^1^H NMR (400 MHz, CDCl_3_) δ: 1.47–1.45 (t, 3H, *J* = 8.0 Hz, CH_3_), 4.15–4.13 (d, 2H, *J* = 8.0 Hz, CH_2_), 3.72 (d, 1H, HA), 4.04 (d, 1H, HB), 5.51 (d, 1H, HX), 7.96–7.03 (d, 8H, ArH) 9.23 (S, NH). MS (ESI) m/z calcd. for C_17_H_17_N_2_OF: 266.3376, found 266.3345.
*5-(4-bromophenyl)-3-(4-ethoxyphenyl)-4,5-dihydro-1H-pyrazole (**EH8**):*^1^H NMR (400 MHz, CDCl_3_) δ: 1.47–1.45 (t, 3H, *J* = 8.0 Hz, CH_3_), 4.15–4.13 (q, 2H, *J* = 8.0 Hz, CH_2_), 3.76 (d, 1H, HA), 4.02 (d, 1H, HB), 5.51 (d, 1H, HX), 7.89–7.70 (d, 8H, ArH), 9.24(S, NH). C_17_H_17_N_2_OBr: 345.2336, found 345.2340.

### 3.2. Enzyme Assays

MAO activities were assayed as described previously, using recombinant human MAO-A and MAO-B and kynuramine (0.06 mM) and benzylamine (0.3 mM) as substrates, respectively; both substrate concentrations were 1.5 × K_m_ (K_m_ = 0.041 and 0.20 mM, respectively). The enzymes and chemicals were purchased from Sigma-Aldrich (St. Louis, MO, USA) [17].

### 3.3. Analysis of Enzyme Inhibitions and Kinetics

The inhibitory activities of the four compounds against MAO-A and MAO-B were first observed at a concentration of 10 µM. IC_50_ values for MAO-B by compounds showing residual activities of <50% were determined. Kinetic studies were performed on the most potent inhibitors, i.e., **EH6** and **EH7** for MAO-A and MAO-B, at five concentrations of the substrates and three inhibitor concentrations, as previously described [18].

### 3.4. Analysis of Inhibitor Reversibilities

Reversibilities of **EH7** were analyzed using a dialysis method after preincubating with MAO-B for 30 min, as previously described. The concentrations used were **EH7** at 0.15 µM, lazabemide (a reversible MAO-B reference inhibitor) at 0.20 µM, and pargyline (an irreversible MAO-B reference inhibitor) at 0.30 µM. The relative activities for undialyzed (A_U_) and dialyzed (A_D_) samples were compared to determine the reversibility patterns [19].

### 3.5. Molecular Dynamics Study

The 3D structures of MAO-A (PDB ID: 2Z5X and MAO-B: 2V5Z), hereafter referred to as MAO-A and MAO-B, respectively, were retrieved from the Protein Data Bank (PDB) [46,47]. Molecules that were co-crystallized with MAO-A and MAO-B were deleted with the exception of the co-enzyme FAD. Missing residues were filled with the aid of MODELLER tools found in the graphic user interface of Chimera [48,49]. Marvin Sketch was employed to draw the 2D structures of **EH1**, **EH6**, **EH7**, and **EH8**; afterwards, geometrical and structural optimizations of these ligands were carried out using B3LYP/6-311 ++G (d,p) and the UFF forcefield of Gaussian 16, respectively [50]. Active site identification was achieved by centering the grid box around a co-crystallized ligand found on the MAO-A and MAO-B proteins downloaded from PDB. Molecular docking was then carried out using AutoDock Vina [51]. The systems were then subjected to a simulation run of 50 ns using Amber18 software (San Francisco, CA, USA) [52]. Eight systems were set up for simulation (MAO-A:**EH1**, MAO-A:**EH6**, MAO-A:**EH7**, MAO-A:**EH8**, MAO-B:**EH1**, MAO-B:**EH6**, MAO-B:**EH7**, and MAO-B:**EH8**). The simulation process was carried out in accordance with an in-house protocol. The CPPTRAJ module of Amber18 software was used to analyze the trajectories emanating from the simulation process [53,54].

### 3.6. Target Predicition and ADME Analysis

The biological target and ADME predictions of the lead molecule of **EH7** were assessed at http://www.swisstargetprediction.ch (access on 23 May 2021) [55,56].

## 4. Conclusions

A series of four halogenated pyrazolines with ethoxylated heads (**EH1**, **EH6**, **EH7**, and **EH8**) was synthesized from their corresponding chalcones. All four compounds were explored for their MAO-A and MAO-B inhibitory activities. On the basis of previous studies regarding the role of halogens in various scaffolds with MAO inhibition activities, we mainly focused on the effect of various halogens on the pyrazoline core with the ethoxylated head. According to the results of this study, the synthesized halogenated pyrazolines exhibited good selective MAO-B inhibition. Fluorine-containing pyrazoline (**EH7**) exhibited an improved selectivity profile against the MAO-B isoform. Kinetics and reversibility studies revealed that **EH7** is a selective and competitive inhibitor of MAO-B, with a recovery value similar to that of the reversible reference drug lazabemide. Pyrazolines are said to be optically active compounds, and, from the MM/PBSA values obtained, it can be clearly observed that chirality has minimal influence on the binding of the compound to MAO-B. It may be suggested that the absence of bulky groups on the N1 position of pyrazoline limited the role of chirality in the binding mode of the lead molecule in the current study.

## Figures and Tables

**Table 1:** Inhibitions of recombinant human MAO enzymes by compounds of the **EH** series ^a^.

Compounds	Residual Activity at 10 µM (%)			IC_50_ (µM)			SI ^b^
	MAO-A	MAO-B	AChE	MAO-A	MAO-B	AChE	
**EH1**	99.1 ± 0.27	33.7 ± 0.96	81.0 ± 6.21	>40	5.38 ± 0.16	>40	>7.43
**EH6**	68.1 ± 0.18	19.4 ± 1.01	63.5 ± 4.99	22.3 ± 0.12	0.40 ± 0.051	>40	55.8
**EH7**	46.9 ± 1.25	4.03 ± 0.53	74.0 ± 0.71	8.38 ± 0.88	0.063 ± 0.0042	>40	133.0
**EH8**	33.5 ± 0.65	11.3 ± 0.13	53.7 ± 2.15	4.31 ± 0.013	0.69 ± 0.017	10.8 ± 0.15	6.3
Toloxatone				1.08 ± 0.0025	-	-	
Lazabemide				-	0.11 ± 0.016	-	
Clorgyline				0.0070 ± 0.00070	-	-	
Pargyline				-	0.14 ± 0.0059	-	
Tacrine				-	-	0.27 ± 0.019	

^a^ Results are expressed as the means ± standard errors of duplicate or triplicate experiments. ^b^ SI values are expressed for IC_50_ of MAO-B/IC_50_ of MAO-A. Values for reference compounds were determined after preincubation for 30 min with enzymes.

**Table 2:** BBB assay of standard and **EH** compounds.

Compounds	Bibliography Pe (×10^−6^ cm/s) ^a^	Experimental Pe (×10^−6^ cm/s)	Prediction
**Progesterone**	9.3	9.02 ± 0.11	CNS+
**Verapamil**	16.0	15.53 ± 0.24	CNS+
**Piroxicam**	2.5	2.43 ± 0.30	CNS+/−
**Lomefloxacin**	1.1	1.12 ± 0.01	CNS−
**Dopamine**	0.2	0.22 ± 0.01	CNS−
**EH1**		10.34 ± 0.33	CNS+
**EH6**		14.26 ± 0.80	CNS+
**EH7**		14.13 ± 0.71	CNS+
**EH8**		14.56 ± 0.26	CNS+

CNS+ (high): Pe (10^−6^ cm/s) > 4.00; CNS− (low): Pe (10^−6^ cm/s) < 2.00; CNS± (uncertain): Pe (10^−6^ cm/s) from 4.00 to 2.00. ^a^ From [40].

**Table 3:** Average RMSD, RMSD_AS, RMSF, and IC_50_ of the Apo and bound systems.

Complexes	RMSD	RMSD_AS	RMSF	IC_50_ (µM)
**MAO-A_EH1**	3.35 (0.73)	1.93 (0.18)	1.47 (1.28)	>40
**MAO-A_EH6**	2.63 (0.72)	1.44 (0.27)	1.15 (1.04)	22.3
**MAO-A_EH7**	2.74 (0.59)	2.09 (0.29)	1.07 (0.96)	8.38
**MAO-A_EH8**	3.26 (0.74)	1.96 (0.34)	1.40 (1.28)	4.31
**MAO-B_EH1**	2.29 (0.34)	2.85 (0.37)	0.88 (0.59)	5.38
**MAO-B_EH6**	1.61 (0.22)	1.54 (0.14)	0.83 (0.60)	0.40
**MAO-B_EH7**	1.49 (0.26)	1.60 (0.16)	0.68 (0.72)	0.063
**MAO-B_EH8**	1.79 (0.24)	1.70 (0.25)	0.75 (0.61)	0.69

**Table 4:** The total free binding energy contributions of **EH**, **EH6**, **EH7**, and **EH8**.

Complexes	Energy (kcal/mol)						
	∆E_vdW_	∆E_ele_	∆G_gas_	∆G_GB_	∆G_SA_	∆G_sol_	∆G_bind_
MAO-B**_EH1**	−6.68	−34.66	−35.32	35.91	−1.68	35.74	−4.11
	(±0.19)	(±4.28)	(±4.20)	(±3.99)	(±0.01)	(±3.98)	(±0.36)
MAO-B**_EH6**	−25.46	−94.22	−96.77	108.35	−4.28	107.92	−11.15
	(±0.15)	(±2.01)	(±2.06)	(±2.64)	(±0.01)	(±2.63)	(±0.88)
MAO-B**_EH7**	−36.79	−32.46	−85.96	55.09	−5.12	49.67	−36.29
	(±0.15)	(±0.54)	(±0.53)	(±0.50)	(±0.01)	(±0.50)	(±0.13)
MAO-B**_EH8**	−36.79	−101.78	−105.46	106.54	− 5.35	106	−5.48
	(±0.15)	(±1.73)	(±1.74)	(±1.56)	(±0.01)	(±1.56)	(±0.37)
MAO-A**_EH1**	−33.17	−12.98	−13.31	−12.85	−4.65	12.81	−5.06
	(±0.22)	(±4.07)	(±4.20)	(±2.86)	(±0.01)	(±2.85)	(±1.49)
MAO-A**_EH6**	−36.75	−14.50	−14.88	13.78	−4.86	13.73	−11.33
	(±0.18)	(±2.36)	(±2.39)	(±1.61)	(±0.01)	(±1.61)	(±0.97)
MAO-A**_EH7**	−32.75	−13.21	−13.54	−36.79	−4.85	13.21	−32.55
	(±0.15)	(±2.06)	(±2.06)	(±0.15)	(±0.01)	(±1.36)	(±1.07)
MAO-A**_EH8**	−7.52	−12.07	−12.14	12.92	−2.61	12.89	−7.48
	(±0.15)	(±2.49)	(±2.56)	(±2.30)	(±0.01)	(±0.15)	(±0.15)

**Table 5:** The differential free binding energy of MAO-B_**EH7**R and MAO-B_**EH7**S enantiomers.

	Energy (kcal/mol)						
Complexes	∆E_vdW_	∆E_ele_	∆G_gas_	∆G_GB_	∆G_SA_	∆G_sol_	∆G_bind_
MAO-B**_EH7**R	−36.79	−32.46	−85.96	55.09	−5.12	49.67	−36.2
	(±0.15)	(±0.54)	(±0.53)	(±0.50)	(±0.01)	(±0.50)	(±0.13)
MAO-B**_EH7**S	−53.42	−17.59	−22.93	19.88	−5.62	19.31	−36.17
	(±0.08)	(±1.56)	(±0.50)	(±1.49)	(±0.01)	(±0.47)	(±0.12)

## Sample Availability

Samples of the compounds are available from the authors.
